# Quantitative High-Throughput, Real-Time Bioassay for Plant Pathogen Growth *in vivo*

**DOI:** 10.3389/fpls.2021.637190

**Published:** 2021-02-10

**Authors:** Chunqiu Zhang, Ben N. Mansfeld, Ying-Chen Lin, Rebecca Grumet

**Affiliations:** ^1^Key Laboratory of Biology and Genetic Improvement of Horticultural Crops, Beijing Key Laboratory of Vegetable Germplasm Improvement, National Engineering Research Center for Vegetables, Beijing Academy of Agriculture and Forestry Sciences, Beijing, China; ^2^Graduate Program in Plant Breeding, Genetics and Biotechnology, Department of Horticulture, Michigan State University, East Lansing, MI, United States

**Keywords:** *Phytophthora capsici*, quantitative bioassay, cucumber, pepper, early pathogen growth

## Abstract

Effective assessment of pathogen growth can facilitate screening for disease resistance, mapping of resistance loci, testing efficacy of control measures, or elucidation of fundamental host-pathogen interactions. Current methods are often limited by subjective assessments, inability to detect pathogen growth prior to appearance of symptoms, destructive sampling, or limited capacity for replication and quantitative analysis. In this work we sought to develop a real-time, *in vivo*, high-throughput assay that would allow for quantification of pathogen growth. To establish such a system, we worked with the broad host-range, highly destructive, soil-borne oomycete pathogen, *Phytophthora capsici*. We used an isolate expressing red fluorescence protein (RFP) to establish a microtiter plate, real-time assay to quantify pathogen growth in live tissue. The system was successfully used to monitor *P. capsici* growth *in planta* on cucumber (*Cucumis sativus*) fruit and pepper (*Capsicum annuum*) leaf samples in relation to different levels of host susceptibility. These results demonstrate usefulness of the method in different species and tissue types, allowing for highly replicated, quantitative time-course measurements of pathogen growth *in vivo*. Analyses of pathogen growth during initial stages of infection preceding symptom development show the importance of very early stages of infection in determining disease outcome, and provide insight into points of inhibition of pathogen growth in different resistance systems.

## Introduction

Measurement of pathogen growth on plant tissue is an important tool in developing disease management strategies. It can facilitate phenotyping to identify resistance loci, test efficacy of control measures, or elucidate fundamental host-pathogen interactions. Traditional methods to measure disease response, such as calculation of host survival rates, scoring of visual symptoms, proportion of plants exhibiting symptoms, or symptom severity provide useful information about disease progression. While effective and useful in the field, these visual approaches have limited accuracy, require training, and can be affected by inherent bias of the researcher (Ward et al., 2004; Jain et al., 2015). In recent years measurement of disease progression has been enhanced by the use of high-throughput digital imaging such as thermography, fluorescence imaging and hyperspectral techniques for quantitative analysis of host responses in a variety of host-pathogen systems (Fang and Ramasamy, 2015; Mutka and Bart, 2015; Laflamme et al., 2016; Mahlein et al., 2019). These phenotype-based methods are informative, but also have limitations. They are not able to detect pathogen growth prior to the occurrence of symptoms; depending on the phenotype scored, they may not be pathogen-specific; and disease symptoms may not reflect extent of pathogen growth, especially in cases of tolerance vs. resistance (Ward et al., 2004; Rousseau et al., 2013). Therefore, a reliable method for assessment of pathogen growth in live plant tissue is of importance.

Quantification of pathogen growth typically relies upon the ability to measure pathogen-specific biochemical components, proteins, or nucleic acid sequences (Ward et al., 2004; Vincelli and Amsden, 2013). If pathogens produce unique compounds such as ergosterol or chitin that are not otherwise present in the host, pathogen biomass can be determined by measuring those compounds. Such compounds, however, do not provide specificity among pathogens. Immunological methods detecting pathogen-specific epitopes can quantify pathogen growth, and have been extensively used, especially for viral diagnostics (Ward et al., 2004). However, immunological approaches can be limited by ability to obtain antibodies that exhibit the required specificity for the pathogen in question. More broadly applicable PCR-based methods, including recent techniques such as recombinase polymerase amplification and loop-mediated isothermal amplification, that target species- or isolate-specific marker sequences have been increasingly applied to quantify amount of pathogen present in a sample (Vincelli and Amsden, 2013; Donoso and Valenzuela, 2018). While all of the above methods can be reliable and sensitive, they are also destructive, and so can only provide information from a single time point.

A non-destructive approach that can facilitate real-time monitoring of specific pathogen growth is the use of reporter genes, such as genes encoding fluorescent proteins, often in combination with microscopy to observe the pathogen infection process and location. This approach has been used in numerous fungal, oomycete and bacterial plant pathogen systems (e.g., Chen et al., 2003; Bloemberg, 2007; Dunn et al., 2013; Hupp et al., 2019). Prior studies in our lab utilized fluorescent strains of *Phytophthora capsici* developed by Dunn et al. (2013) to observe pathogen growth on cucumber fruit samples via fluorescence microscopy, and in a microtiter assay to test for effect of aqueous and methanolic extracts from cucumber fruit peel on pathogen growth (Ando et al., 2015; Mansfeld et al., 2020). While microscopy allows for observations *in vivo*, quantitative assessment and multiple replications, if possible, are labor- and time-intensive. The microplate experiments provided quantitative analyses over time in culture conditions, but not in live plant tissue.

In the current work we sought to overcome limitations of the above methods by developing a real-time, high-throughput assay to quantify pathogen growth in live tissue. To establish such a system, we worked with the highly destructive, soil-borne oomycete pathogen, *P. capsici*, which was first identified in pepper, has a broad host range causing severe damage in many solanaceous, cucurbitaceous, and legume crops (Granke et al., 2012; Lamour et al., 2012; Sanogo and Ji, 2012). The primary source of inoculum in the growing season is zoospores which are spread in the field via water, such as rainfall or irrigation (Granke et al., 2012). Upon reaching the plant surface, zoospores lose their flagella and encyst; germ tubes then emerge from the zoospore and form appressoria which allow for direct penetration into the plant cells (Lamour et al., 2012). *P. capsici* causes different symptoms depending on the crop, and can infect various tissue types and life stages. Pepper (*Capsicum annuum*) plants can be infected at all growth stages, and roots, stems, foliage and fruit are all susceptible. Symptoms include damping off, stunted growth, wilting, and in severe cases, death (Lamour et al., 2012; Sanogo and Ji, 2012). For cucumber (*Cucumis sativus*), very young fruit are most susceptible relative to other parts of the plant such as leaves and vines which remain largely unaffected (Gevens et al., 2006). Visible symptoms first occur as water soaking on the fruit surface 2–3 days post inoculation followed by extensive sporulation and tissue collapse (Gevens et al., 2006; Ando et al., 2009; Colle et al., 2014). While sensitive, quantitative systems have been established to measure *P. capsici* [e.g., PCR-based protocols (Silvar et al., 2005; Lan et al., 2013)], they are typically destructive and do not have the capacity to monitor pathogen growth *in planta* in real time.

Establishment of a method to obtain extensively replicated, high-resolution data also can enable quantification and statistical analysis of pathogen growth parameters. For example, the application of parametric non-linear growth curve models, which typically follow sigmoidal functions, can allow accurate modeling and prediction of microbial growth (Zwietering et al., 1990). Models such as the Gompertz model, which has been extensively used for microbial, food and cancer growth modeling (Tjørve and Tjørve, 2017), can enable the researcher to identify the effects of a treatments and/or genotypes on parameters such as lag time, maximum growth rate and maximum pathogen abundance.

In this study we use a *P. capsici* isolate expressing red fluorescent protein (RFP) *tdTomato* gene (Dunn et al., 2013) to establish a microtiter plate, real-time assay to quantify pathogen growth in live tissue. We then use this system to monitor *P. capsici* growth *in planta* on cucumber fruit and pepper leaf samples in relation to different levels of host susceptibility. These results demonstrate usefulness of the method in different species and tissue types. This method allows for highly replicated, quantitative time-course measurements of pathogen growth *in vivo* during initial stages of infection preceding symptom development and shows the importance of very early stages of infection in determining disease outcome in these systems. In addition, the highly replicated data are suitable for growth curve modeling to facilitate hypothesis development regarding key aspects of pathogen response.

## Materials and Methods

### Plant Material

Three cucumber cultigens were used for these experiments: susceptible pickling cucumber breeding line, “Gy14” (originally obtained from the University of Wisconsin and multiplied in the greenhouse); the slicing cucumber cultivar, “Poinsett 76” (Seedway, LLC, Hall, NY, United States), with age-related resistance (ARR) to *P. capsici*; and the doubled haploid breeding line “A4-3,” with moderate young-fruit resistance to *P. capsici* “A4-3” was derived from breeding line MSU 109483-5-3, a self-pollinated line from cucumber PI109483 (Grumet and Colle, 2017). The doubled haploid line was generously produced by Rijk-Zwaan, Netherlands. For the ARR experiments, hand-pollinated fruits were produced on plants in the greenhouse under growing conditions as described by Ando et al. (2015). Fruits of “Poinsett 76,” which exhibits ARR (Mansfeld et al., 2020) were harvested at 8 vs. 16 days post pollination (dpp); 16 dpp fruit were used for comparisons of “Poinsett 76” (ARR+) and “Gy14” (ARRı-). For the comparisons of young fruit from “Gy14” and “A4-3,” trellised plants were grown in the field and pollinated by bees under growing conditions as described by Grumet and Colle (2017). Once the period of fruit set began, young fruits were harvested at 5–7 dpp as assessed by fruit size.

Two pepper cultigens were used: the susceptible cultivar “ACE” (Johnny’s Selected Seeds; Fairfield, ME, United States), and the resistant breeding line Criollo de Morelos-334 (“CM-334”) (Walker and Bosland, 1999), generously provided by Dr. Paul Bosland (New Mexico State University). Pepper plants were grown in the greenhouse under the same conditions as cucumber. Fifteen six-leaf stage seedlings were used for each repetition of each application. A pair of young, fully expanded leaves was taken from each plant.

### Pathogen Preparation

Zoospore suspensions were prepared from *P. capsici* isolate NY0664-1 expressing RFP gene *tdTomato*, kindly provided by C. Smart, Cornell University (Dunn et al., 2013). This isolate was originally obtained from pepper as described by Dunn et al. (2013) and also has been used for previous experiments with cucumber (e.g., Mansfeld et al., 2020). The pathogen was cultured on V8 agar medium as described by Mansfeld et al. (2017). After 7 days, the plates were flooded with 10 mL of sterile distilled water to release zoospores. The plates were then incubated at 4 C for 30 min, followed by a 30 min incubation at 25 C to promote zoospore release. The zoospore suspensions were collected by pipet and two 10 μL aliquots of zoospore suspensions were quantified using a Countess cell counter (Invitrogen). The mean concentration was used for subsequent dilutions; zoospore concentrations were quantified again after dilution.

### Fluorescence Microscopy

Preliminary fluorescent microscopy of infection was performed using an EVOS FL Auto imaging system (Thermo Fisher Scientific). Excised cucumber peels of susceptible “Gy14” were affixed to the lid of a 100 mm petri dish using petroleum jelly (Covidien, Mansfield, MA, United States) and inoculated with 10 μL of 5 × 10^5^ zoospores/ml. The petri dishes were sealed with parafilm and carefully inverted and placed on the microscope stage. Samples were observed at 40× magnification and images were captured every 30 min for 48 h.

### Disease Response Tests on Whole Fruit or Leaves

Harvested cucumber fruits and pepper leaves were washed and surface sterilized as previously described (Colle et al., 2014). Young cucumber fruit were inoculated with two, 30 μL droplets of 1 × 10^4^ zoospores/mL of *P. capsici* isolate NY 0664-1-RFP (as per Colle et al., 2014); fruits for ARR experiments were inoculated with two, 30 μl droplets of 1 × 10^5^ or 5 × 10^5^ zoospores/ml (as per Mansfeld et al., 2020). Fruit were then incubated for 5 days at room temperature (22 C) in covered plastic trays lined with wet paper towels, to maintain high humidity as described by Colle et al. (2014). Young fully-expanded pepper leaves were placed into petri dishes with filter paper; 2 ml ddH_2_O was added to each plate to maintain a high relative humidity (>70%) and incubated at room temperature. A 5 μL droplet of 2 × 10^5^ zoospores/mL suspension was placed on the adaxial side of the leaf on each side of the midvein as per Monroy-Barbosa and Bosland (2010). Control leaves were equivalently inoculated with an equal volume of sterile ddH_2_O. A total of 15 plants of each accession were used per replication. Two mature leaves per plants were selected for inoculation. The experiment was replicated three times. The disease level was evaluated on a 5 point scale according to Monroy-Barbosa and Bosland (2010): 0 = no symptoms; 1 = small necrotic tissue with defined borders (associated with hypersensitive response); 2 = dark green, water-soaked lesion; 3 = 15 to 49% of leaf area is wilted, scalded, or necrotic; 4 = 50% or more of the leaf is wilted, scalded, or necrotic; and 5 = 100% of the leaf is necrotic. The leaves were observed for symptom development every day for 5 days post-inoculation. Disease ratings were taken at 2, 3, and 5 dpi. disease severity index was calculated according to the following formula: Σ(number of leaves × scale value)/total number of leaves. Disease severity data were analyzed by analysis of variance.

### Development of the Fluorescent-Based Real-Time High-Throughput Assay

#### Sample Preparation

For cucumber fruit, samples were prepared from the central portion of the fruit using a 6 mm biopsy punch (VWR, Philadelphia, PA, United States). Samples were cut to 5–6 mm thickness, and placed with external surface to the top, into a 96-well (6.5 mm diameter wells), flat bottom, black Greiner microplate (#655086; Greiner Bio-One International, Kremsmunster, Austria). The bottom and sides of the wells were coated with petroleum jelly prior to placement of the samples. For pepper leaves, two disks, one from the center of either side of the midvein, were sampled from each leaf with a 6 mm biopsy punch. Leaf samples were placed adaxial side up into wells containing 150 μL 6% agar (Difco, Detroit, MI, United States).

#### Inoculation Conditions

Experimental conditions were established empirically to ensure consistent infection while preventing contact of inoculum with cut surfaces of the different sample types. Cucumber fruit and pepper leaf samples were tested with varying droplet sizes (3, 5, and 8 μL) and zoospore concentrations (1 × 10^5^, 5 × 10^5^, and 1 × 10^6^ zoospores/mL). Zoospore concentrations within the droplets were chosen to provide equivalent inoculum pressure as used for intact fruits or leaves as determined in our prior experiments or from the literature (Monroy-Barbosa and Bosland, 2010; Colle et al., 2014; Mansfeld et al., 2017).

#### Fluorescence Detection

Florescence was measured by the Tecan Spark^®^ multimode microplate reader (Tecan Group Ltd., Mannedorf, Switzerland). Black, flat-bottom microplates were used to minimize background signals and fluorescence. The excitation, emission wavelength, and bandwidth settings to maximize signal to background were optimized using the pathogen. The following optimized process was used for detection of fluorescence: excitation wavelength = 536 nm, emission wavelength = 586 nm, both excitation and emission bandwidth = 20 nm, number of flashes = 30, integration time = 40 μs, top reading. For each sample well, multiple points were recorded using the optimal read function to ensure that the full surface of the sample was measured. Unless stated otherwise, the maximum detected relative fluorescence units (RFU) within each well was used at each timepoint. To verify suitability of the fluorescence emission intensity for linearity of response and quantification of relative pathogen levels, a range of pathogen concentration was tested using a dilution series of 5 × 10^4^, 1 × 10^5^, 2 × 10^5^, 2.5 × 10^5^, 4 × 10^5^, 4.5 × 10^5^, 5 × 10^5^, 6 × 10^5^, 6.5 × 10^5^, and 1 × 10^6^ zoospores/mL. The dilution series test was repeated three times. Each bioassay experiment included a sample that was inoculated 2 days in advance to calibrate the detection range. The *Z* position was set automatically for each plate based on the control pathogen well.

### Pathogen Growth Bioassays

For the cucumber young fruit comparison, six fruits of “A4-3” and “Gy14” were used for each experiment. Eight samples were taken from each fruit. Six of the samples were inoculated with 3 μl 1 × 10^5^ zoospores/mL *P. capsici;* the other two samples received 3 μl sterile ddH_2_O as a control. For “Poinsett 76” at 8 dpp vs. 16 dpp, and “Poinsett 76” at 16 dpp vs. “Gy14” at 16 dpp, three fruits were harvested for each genotype/age for each experiment. Sixteen samples were taken from each fruit; twelve were inoculated with 5 μl 1 × 10^6^ zoospores/mL *P. capsici*, the other four samples received 5 μl ddH_2_O.

For the pepper “CM-334” and “ACE” comparison, 12 plants were used for each experiment for each line. Two young, fully expanded leaves were taken from each plant, one sample from each side of the leaf as described above. For every column of the microplate, six samples were inoculated with 5 μl *P. capsici* 2 × 10^5^ zoospores/mL and two samples inoculated with 5 μl ddH_2_O as control. Fluorescence measurements were taken for each well every hour for the first 24 or 48 h post inoculation (hpi). Each experiment was repeated three times.

### Statistical Analysis and Growth Modeling

After recording the data, the highest value from each well was used for further analysis. Data were analyzed using R and Origin 8.5 (1991–2010, OriginLab, United States) and plots were made using the ggplot2 and Tidyverse packages in R studio (2009–2018, Version 1.1.463, MathSoft, New Zealand). The relationship between the concentration of the pathogen and the relative intensity of the fluorescence was drawn by Origin 8.5.0 Software. Gompertz growth models were fit to maximum RFU values for each well and modeled using the *all_growthmodels()* and the *grow_gompertz3()* functions in the *growthrates* R package (Petzoldt, 2019). For each experiment, initial estimates for ranges of parameters (*y0* – initial abundance value, *mumax* – maximum growth rate, *K* – maximum abundance, *lambda* – time of lag phase) were determined by examining data for the full plate. For the pepper leaf experiment, the parameter values were set as *y0* = 1000, *mumax* = 0.15, *K* = 10000, *lambda* = 12 for start parameters and an upper bound of *y0* = 3000, *mumax* = 0.5, *K* = 15000, *lambda* = 24. A value of 0 was set to all parameters as a lower bound, to avoid non-biologically relevant predictions. The results for each well were returned and the mean and standard error were calculated per treatment and genotype. Results were plotted using ggplot2. Parameter estimate means were compared using Wilcoxon Signed Rank Test.

## Results and Discussion

### Disease Progression on Cucumber Fruit and Visualization of Growth of *P. capsici* by Fluorescence Microscopy

**FIGURE 1:**
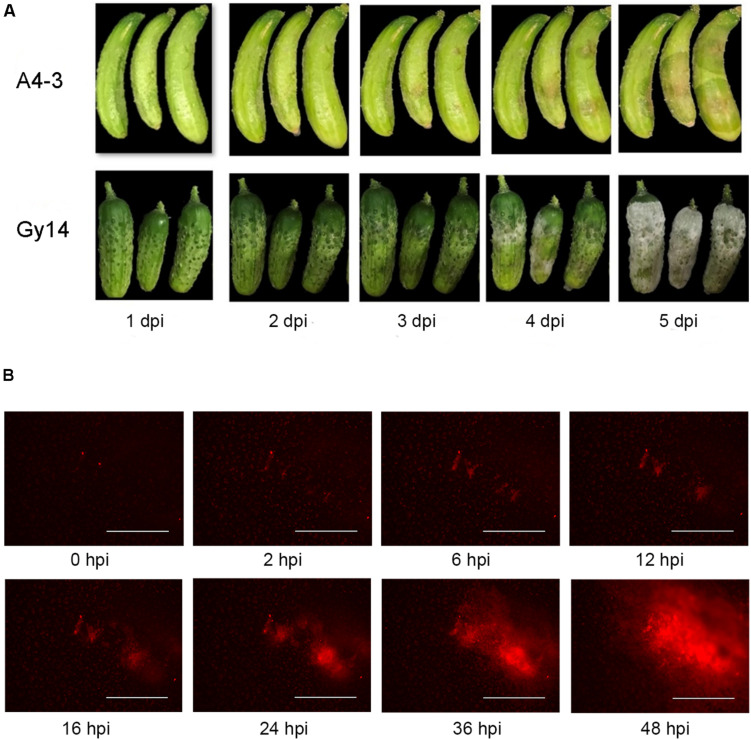
Response of cucumber fruit to inoculation with *P. capsici* isolate NY 0664-1-RFP. **(A)** Symptom development on young fruit (5–7 days post-pollination) of moderately resistant “A4-3” and susceptible “Gy14” breeding lines 1–5 days post inoculation (dpi). **(B)** Fluorescence visualization of growth of *P. capsici* on young “Gy14” fruit. Magnification = 40×; bar = 1,000 μm.

Water soaking symptoms were visible on young cucumber fruit 2–3 days after inoculation with *P. capsici* (Figure 1A). The disease progressed more rapidly on the susceptible “Gy14.” White sporangia were apparent by 4 dpi and completely covered the fruit surface by 5 dpi. To be able to monitor pathogen growth prior to appearance of symptoms, fluorescence microscopy was performed using an EVOS FL Auto imaging system (Figure 1B); images were captured every 30 min for 48 h. As observed by the red fluorescence, the pathogen grew slowly for the first 12 h, after which there was a strong increase in area and intensity of the fluorescence.

### Establishment of a Microtiter Plate Assay for Pathogen Growth *in planta*

Based on the observations via microscopy, we sought to develop a fluorescence-based method that would provide replicated, quantitative measurement of rate of growth and facilitate experimental comparisons between different treatments or genotypes during the initial period post inoculation. Factors important for establishment of the protocol as described below, include sample preparation, inoculation conditions, and detection method.

#### Sample Preparation and Inoculation

**FIGURE 2:**
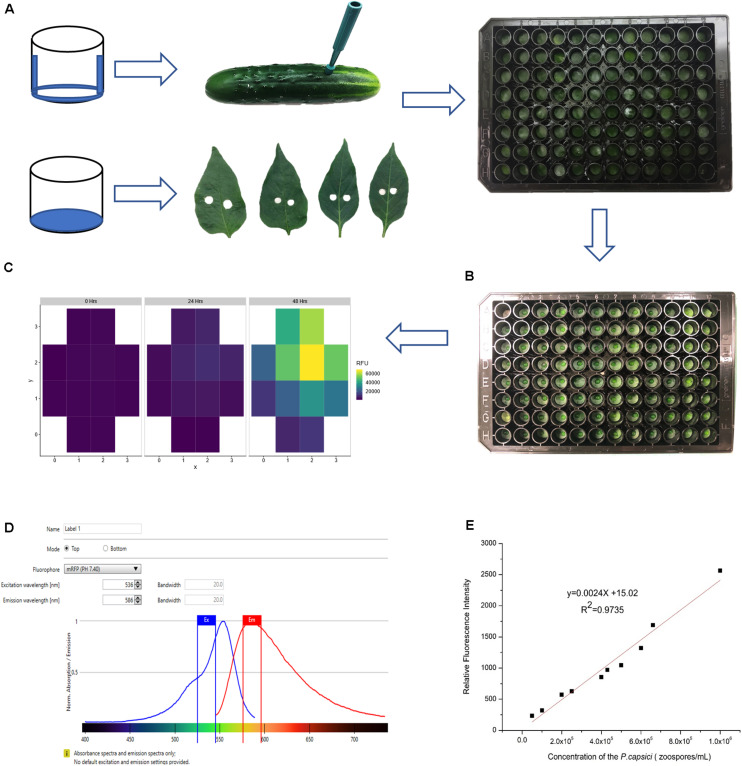
Establishment of conditions for quantitative fluorescence assay. **(A)** Sample preparation for cucumber fruit and pepper leaves. Dehydration was minimized by coating the bottom and sides of the wells with petroleum jelly (fruit samples) or adding 150 μL 6% agar to each well (leaf samples). **(B)** Inoculum droplet size was minimized to prevent the pathogen from contacting the cut surface of samples. **(C)** Multiple fluorescence readings were taken from each well. **(D)** Testing gradient of wavelengths to optimize excitation and emission wavelengths. **(E)** Serial dilution of *P. capsici* NY 0664-1-RFP and the relative fluorescence intensity (5 × 10^4^, 1 × 10^5^, 2 × 10^5^, 2.5 × 10^5^, 4 × 10^5^, 4.5 × 10^5^, 5 × 10^5^, 6 × 10^5^, 6.5 × 10^5^, 1 × 10^6^ zoospores/mL). Each point is the mean of three replicate samples.

Important considerations for sample preparation and inoculation include minimizing dehydration of tissue samples and preventing contact between cut edges of the sample and the inoculum. Care was taken to prevent wounding the upper surface when removing samples from the punches. Fruit samples were oriented in the well with external surface to the top; leaf samples were oriented with adaxial surface to the top. For the fruit samples, the bottom and sides of the wells were coated with petroleum jelly to prevent sample dehydration during the experimental period (24–48 h) (Figure 2A). For leaf samples, hydration was maintained by adding agar to the base of each well. All plates were covered with plate lids throughout the experiment.

The primary mode of infection by *P. capsici* is via appressorial-mediated penetration, however, *P. capsici* also can infect via wound sites, and in doing so, overcome host resistances (Krasnow et al., 2014; Ando et al., 2015; Alzohairy et al., 2020). To prevent the inoculum from contacting the cut surfaces of the tissue samples, it was essential to use very small volumes applied to the center of the sample (Figure 2B). Testing different droplet sizes (3, 5, and 8 μL) showed that 5 μL was the maximum volume that can be used for inoculation without sliding off the sample surface. All experiments were performed with either 3 or 5 μL zoospore suspensions. To ensure reproducible infection, each droplet size was tested with a set of three inoculum concentrations, 1 × 10^5^, 5 × 10^5^, and 1 × 10^6^ zoospores/ml. The droplet size and concentration needed to ensure consistent infection of susceptible samples in each experiment were determined empirically by prior testing for each plant material and condition in question (e.g., cucumber, pepper; fruit, leaf; fruit age).

#### Fluorescence Process Determination

The excitation, emission wavelength and bandwidth setting to maximize signal relative to background were optimized using zoospores suspended in water. The excitation and emission wavelengths tested were based on values for isolated RFP encoded by *tdTomato* (554 and 581 nm, respectively; Carroll et al., 2010) and used by Dunn et al. (2013) when expressed in *P. capsici* isolate NY0664-1 (530 and 590 nm, respectively). A gradient of wavelengths 530–550 nm for excitation and 580–590 nm for emission showed that the optimum excitation and emission wavelength in our conditions were 536 nm and 586 nm, respectively (Figure 2D). Equivalent results were obtained using zoospores suspended in water, pathogen growing on agar, or pathogen on fruit surface. The zoospore dilution curve (Figure 2E) showed that the intensity of fluorescence was highly correlated with pathogen concentration (*R*^2^ = 0.9735), indicating that this method can be used to quantify relative pathogen levels.

The saturation threshold used to amplify and calibrate fluorescence signals was based on maximum detected fluorescence at the first measured timepoint. Thus, if the gain is based on a sample with low fluorescent signal, it could quickly become saturated and uninformative as the pathogen grows. To avoid this problem and maximize signal detection over time, we included a sample with high fluorescence at time-point zero, using either fluorescent mycelia-covered agar plugs, or fruit samples inoculated 2 days prior to the experiment. Timepoint zero gain thresholds set on either of those options successfully allowed for maximum detection without saturation as the fluorescence of freshly inoculated experimental samples increased throughout the experiments.

**FIGURE 3:**
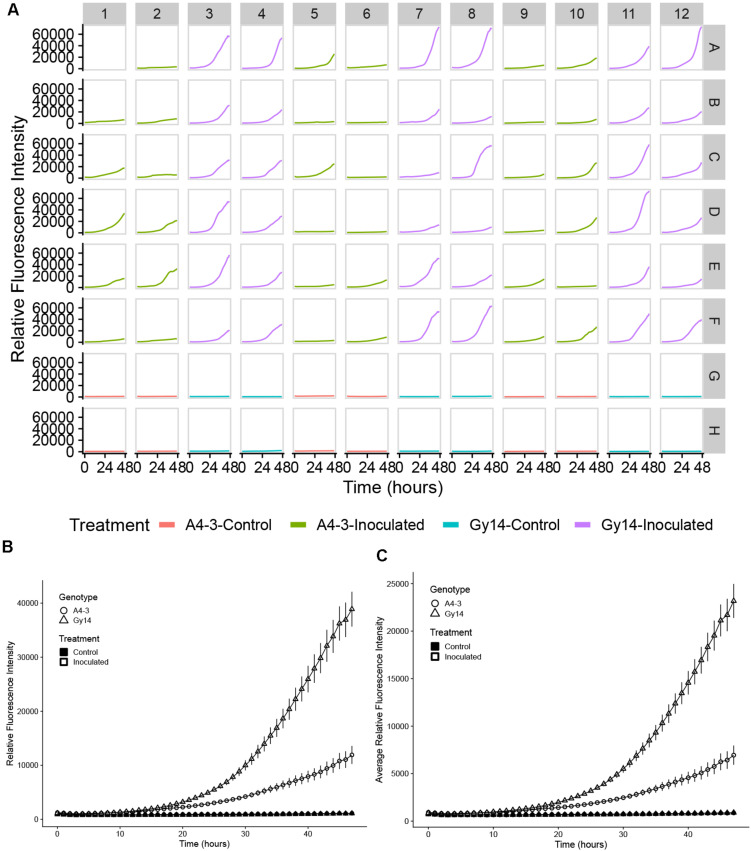
Quantification of growth of *P. capsici* NY 0664-1-RFP on samples from young fruit (5–7 days post pollination) of “A4-3” (moderately resistant) and “Gy14” (susceptible) cucumber breeding lines. **(A)** Time-lapse fluorescence readings of each sample for the first 48 h post inoculation. Columns 1, 2, 5, 6, 9, and 10 are samples from the “A4-3” and column 3, 4, 7, 8, 11, and 12 are samples from “Gy14.” Rows A–F were inoculated with 3 μL 1 × 10^5^ zoospores/mL *P. capsici*. Rows G and H were inoculated the same volume of distilled water; samples within a column are from the same fruit. Well A1 was the sample inoculated 2 days before the experiment for calibration. Readings were collected every hour for the first 48 hpi. **(B,C)** Fluorescence intensity from young fruits of “A4-3” and “Gy14.” **(B)** The value for each time point is the mean of maximum values for 36 samples from six fruits for the inoculated samples and the mean of maximum values 12 samples from six fruits for the control samples. **(C)** As for **(B)** except each value is the mean of the mean values for all replicate samples. Error bars represent the standard error. The experiment was repeated three times with equivalent results.

Ability to detect growth of the pathogen on the host surface can be influenced by location within the well. As the initial foci of infection are microscopic, if fluorescence is measured at a single point, early growth might not be detected. To overcome this limitation, emission measurements were performed using the optimal read function, that measures fluorescence at multiple, spatially separated spots optimized to cover the full well as determined by the beam diameter (Tecan Spark Pro Manual). For a 96-well plate, five read positions are recorded for each well (Figure 2C). Figure 2C illustrates heterogeneity of growth on the surface of the sample, indicating the value in scanning the full well. Subsequent data analyses were performed using the highest value in each well at each time point. While the highest well value was selected to represent maximum pathogen growth and increase ability to detect early pathogen growth, equivalent trends were observed if mean values per well were plotted (Figures 3B,C).

### Quantification of Pathogen Growth

#### Infection of Young Cucumber Fruit

Quantification of pathogen growth was performed on samples of young cucumber fruit (5–7 dpp) from the susceptible “Gy14” and moderately resistant “A4-3” breeding lines. Typical of growth patterns showing a lag phase followed by exponential increase, during the first 10–15 hpi, pathogen growth was slow and similar for both “Gy14” and “A4-3” (Figure 3). After that, the growth rate increased dramatically and differed between the two lines. The rate of growth as measured by increase in fluorescence from 15 to 30 hpi, was approximately twice as great for “Gy14” as for “A4-3” (5.18-fold increase for “Gy14” vs. 2.51-fold increase for “A4-3”) (*t*-test, *n* = 36, *P* < 0.001). Furthermore, 11 of the 36 “A4-3” samples essentially had no increase (less than 1.5-fold increase during the time period 15–30 hpi). The remainder exhibited an average increase of 3.7-fold; thus, even those that did enter exponential growth had a significantly lower rate of increase than for “Gy-14” (*t*-test, *P* < 0.001). In contrast, none of the “Gy14” samples increased less than 1.5×. Similar results were observed in all three experiments with 2.06-, 2.00-, and 1.61-fold greater rate of increase on “Gy14” than “A4-3.” No increase in fluorescence was detected in the control.

#### Age-Related Resistance in Cucumber Fruit

**FIGURE 4:**
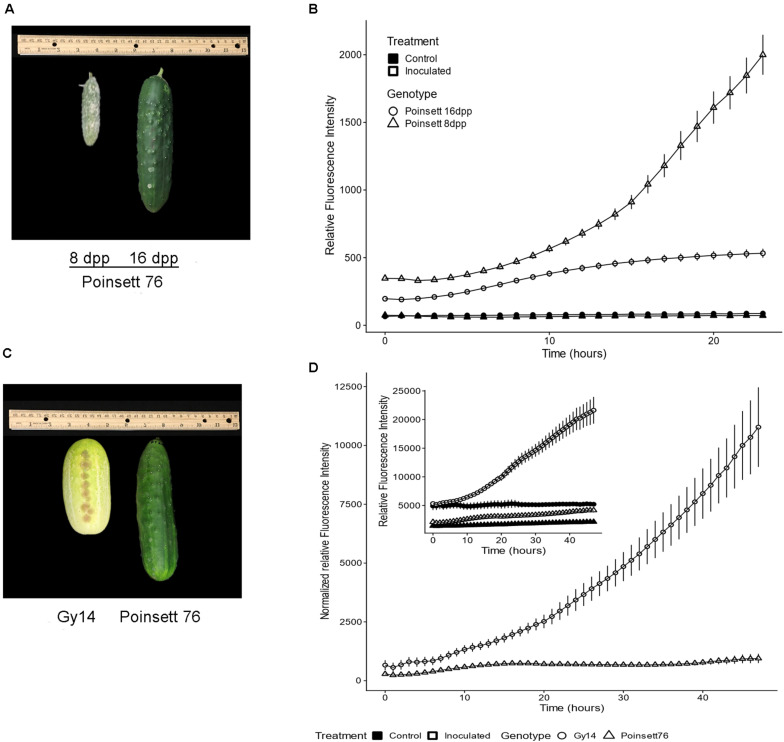
**(A)** Symptom development on “Poinsett 76” fruit at 8 and 16 days post-pollination (dpp). Photograph was taken at 5 days post inoculation. **(B)** Fluorescence intensity curve of *P. capsici* NY 0664-1-RFP on “Poinsett 76” at 8 and 16 dpp. Samples were inoculated with 5 μL 1 × 10^6^ zoospores/mL *P. capsici.* Fluorescence readings were collected every hour. The value for each time point is the mean of 36 samples from six fruits for the inoculated samples and the mean of 12 samples from six fruits for the control samples. Error bars represent standard error. The experiments were repeated three times with equivalent results. **(C)** 16 dpp “Gy14” and “Poinsett 76” fruit showing difference in skin color. Photograph was taken at 5 days post inoculation. **(D)** Fluorescence intensity curve of *P. capsici* on “Gy14” and “Poinsett 76” at 16 dpp. Experimental conditions were as for **(B)**. To adjust for differing baselines between “Gy14” and “Poinsett 76,” the values of control for a given genotype and timepoint were subtracted from the mean value of the corresponding infected samples at each timepoint. Error bars represent standard error. The experiments were repeated three times with equivalent results. The individual sample wells for **(B,D)** are shown in Supplementary Figure 1.

Cucumber fruit can exhibit an ARR to *P. capsici*, wherein young fruit are highly susceptible, but become resistant as they transition away from the period of exponential fruit growth, at approximately 10–12 dpp (Gevens et al., 2006; Ando et al., 2015; Mansfeld et al., 2017). Comparison of samples from fruit of slicing cucumber cultivar “Poinsett 76” at 8 and 16 dpp inoculated with 5 μL 1 × 10^6^ zoospores/ml showed marked increase in pathogen growth on 8 dpp fruit, especially after 10 hpi, increasing ∼3.5-fold from 10 to 24 hpi (Figures 4A,B). On the other hand, pathogen growth on 16 dpp fruits slowed after 10 hpi and essentially stopped by approximately 15 hpi. Similar results were observed in all three experiments with growth largely ceasing in the 16 dpp “Poinsett 76” fruit by 15 hpi in each case. These results are consistent with electron microscopy showing evidence of pathogen death on 16 dpp “Poinsett 76” fruit by 8 hpi (Mansfeld et al., 2020).

Not every cucumber variety exhibits ARR (Mansfeld et al., 2020). Comparison of ARR-expressing “Poinsett 76” at 16 dpp with non-ARR expressing “Gy14” fruits at 16 dpp fruits also showed marked differences in pathogen growth (Figures 4C,D). The 16 dpp “Gy14” fruit samples had baseline fluorescence both in the control and inoculated wells (Figure 4D inset), likely due to their visible differences in epidermal pigmentation (Figure 4C). To adjust for differing baselines, the values for control samples for a given genotype and timepoint were subtracted from the mean value of the corresponding infected samples at each timepoint. The growth rate of the pathogen appeared to be equivalent on the two genotypes until approximately 8 hpi, after which they began to diverge. “Gy14” (ARR-) samples showed a dramatic increase in fluorescence after 10 hpi. For “Poinsett 76” (ARR+), minimal pathogen growth was observed during the 48 h with only 5/36 samples showing increased fluorescence between 10 and 48 hpi (Supplementary Figure 1).

#### Pathogen Growth on Resistant and Susceptible Pepper Lines

**FIGURE 5:**
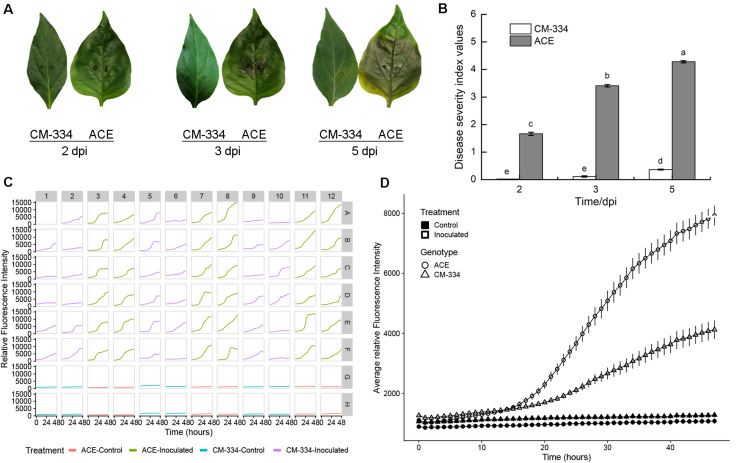
**(A)** Disease progression on pepper leaves of “CM-334” and “ACE” after inoculation with *P. capsici* NY 0664-1-RFP inoculation at 2, 3, and 5 days post inoculation. **(B)** Disease severity over time using the disease rating scale as described in the section “Materials and Methods.” Each value is the mean of three experiments (30 leaves/genotype/experiment). Vertical bars represent standard errors. Data were analyzed by analysis of variance. Bars represented by different letters are significantly different (LSD, *P* < 0.05) **(C)** Time-lapse fluorescence readings of each sample for the first 48 h post inoculation. A pair of young, fully expanded leaves were selected, and two punches were taken from each leaf (one on each side of the midvein, approximately halfway from the leaf base). Columns 1, 2, 5, 6, 9, and 10 are samples from the “CM-334” leaves and columns 3, 4, 7, 8, 11, and 12 are samples from “ACE” leaves. For every row A–F is inoculated with 5 μL 2 × 10^5^ zoospores/mL *P. capsici*. For row G and H the samples were inoculated the same volume of distilled water. Well A1 was the sample inoculated 2 days before the experiment for calibration. **(D)** Fluorescence intensity from young fruits of “CM-334” and “ACE.” Time points were collected every hour within the first 48 hpi. The value of each time point for the inoculation is the mean of 36 samples from 18 leaves, for control is the mean of 12 samples from six leaves. Error bars represent the standard error. The experiment was repeated three times with equivalent results.

We sought to extend this approach to additional species, and importantly, to leaf samples, as that is frequently the tissue studied. Comparisons were made between the susceptible pepper cultivar, “ACE,” and a resistant landrace, Criollo de Morelos-334 (“CM-334”), extensively used in pepper breeding (Walker and Bosland, 1999). Following inoculation of leaves with 5 μL of 2 × 10^5^ zoospores/mL, leaves from the susceptible cultivar “ACE” showed the typical progression of *P. capsici* symptoms, beginning with small, irregular water soaked lesions that increase with time, causing leaves to turn light tan or brown (Barchenger et al., 2018). Water-soaked symptoms appeared at 2 dpi (Figure 5A). At 3 dpi the leaves turned brown around the inoculation site and by 5 dpi brown color had diffused throughout the whole leaf and cracks appeared around the inoculation site. Comparisons of disease severity at 2, 3, and 5 dpi showed progressive disease development on the susceptible “ACE” (Figure 5B). In contrast, leaves of the resistant “CM-334” pepper, showed a low level of disease severity and most, but not all, leaves remained free of symptoms. Leaves treated with distilled water did not exhibit any disease symptoms. The result of fluorescence quantification was consistent with the phenotype, with greater growth on “ACE” than “CM-334” (Figures 5C,D). Initial increases in fluorescent signals were detected at a comparable rate for the first 15 hpi on both sets of samples. After that, the average growth rate and final fluorescence values at 48 hpi were approximately two times greater on “ACE” than on “CM-334.” Examination of individual wells showed that each of the susceptible “ACE” samples exhibited strong increases in fluorescence (Figure 5C). In contrast, 16/36 of the “CM-344” wells showed only minimal increases, suggesting failure to establish infection. The occurrence of “CM-344” samples exhibiting pathogen growth is consistent with the whole leaf assays (Figure 5B) that showed occasional successful infection and delayed rate of disease development.

### Modeling of Pathogen Growth

**FIGURE 6:**
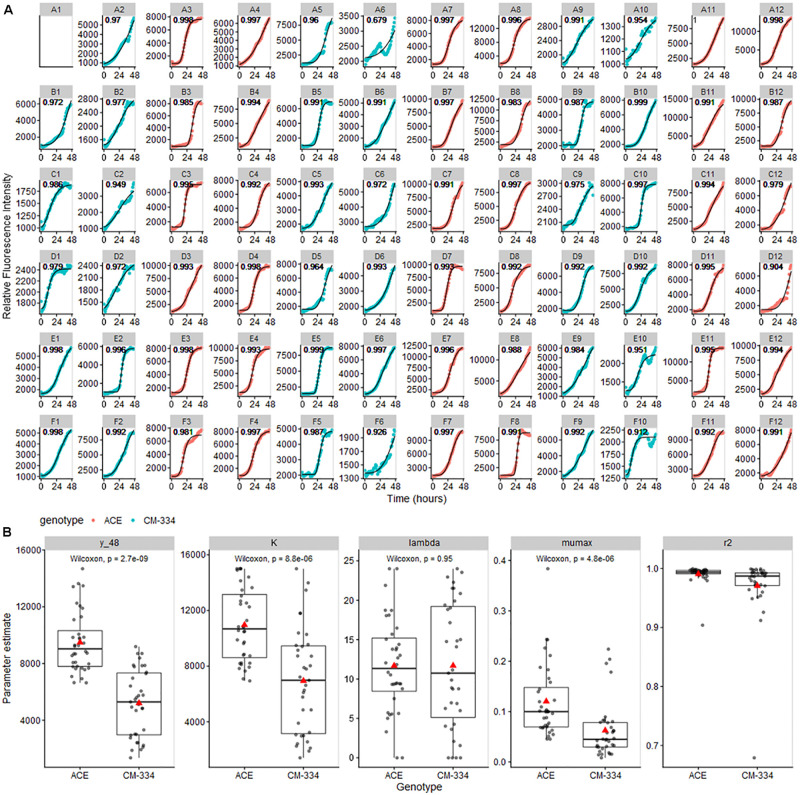
**(A)** Gompertz curves for pathogen growth on pepper leaf samples. Curves were fit to maximum RFU values for each well and modeled using the *all_growthmodels()* function in the growthrates R package (Petzoldt, 2019). Note that scales vary among individual figures to be able to clearly observe the model fit and growth trends in each well. **(B)** Parameter estimate means (predicted value at 48 h (y_48), *K*, maximum abundance; *lambda*, time of lag phase; *mumax*, maximum growth rate) were compared using Wilcoxon Signed Rank Test. Gray lines across boxplots represent the median, red arrows represent the mean.

As a proof of utility for data obtained from our bioassay system, data from the pepper leaf microtiter plate experiments were analyzed using the Gompertz model as implemented in the *growthrates* R package (Petzoldt, 2019). Model fitting yielded high *R*^2^ scores (mean *R*^2^ values, 0.945 and 0.991 for “CM-334” and “ACE,” respectively) indicating that the Gompertz model is appropriate for our pathogen growth data (Figure 6; note that scales vary among individual figures to better show model fit and growth trends in each well) Comparisons of the modeled growth parameters showed a strong reduction in inferred pathogen level at *t* = 48 h, predicted maximum pathogen abundance *(K)*, and maximum growth rate *(mumax)* in the resistant pepper genotype “CM-334” (Figure 6B), There was only a moderate difference in lag phase time *(lambda)* between the two genotypes. These observations suggest that response processes subsequent to the lag phase, contributed to inhibition of disease development in “CM-344.” Model analysis for the cucumber experiments also showed excellent fit (*R*^2^ values 0.945–0.997; Supplementary Figure 2). These analyses indicate that the described bioassay and modeling approaches can help researchers identify crucial stages in host infection and develop biological hypotheses that can further their understanding of infection and resistance processes and distinguish between different potential modes of resistance.

## Conclusion

We have developed a real-time microtiter bioassay to quantitatively measure pathogen growth *in planta* and demonstrated usefulness in different tissue types and species. In each case, samples from susceptible tissues showed significantly greater pathogen growth as measured by fluorescence emissions than those from resistant tissues. These differences were observable very early in the infection process, prior to the appearance of visible symptoms. As many fluorescently labeled pathogen strains have been developed for microscopy studies, this approach should be transferable and broadly applicable to many plant-pathogen systems. Such analyses can provide highly replicated data allowing comparisons across treatment variables such as genotype, tissue type, age, or experimental treatment conditions. The highly replicated data are also amenable to modeling approaches that can provide insight into host pathogen interactions and critical stages influencing success or inhibition of infection.

## Data Availability Statement

The original contributions presented in the study are included in the article/Supplementary Material, further inquiries can be directed to the corresponding author.

## Author Contributions

BM, CZ, and RG conceived the project and wrote the manuscript. CZ and BM developed and performed the microplate bioassay and statistical analyses. BM and Y-CL performed the microscopy experiments. All authors have read and approved the manuscript.

## Conflict of Interest

The authors declare that the research was conducted in the absence of any commercial or financial relationships that could be construed as a potential conflict of interest.

## Supplementary Material

Supplementary Figure 1 Fluorescence intensity curves of *P. capsici* NY 0664-1-RFP on “Poinsett 76” fruit at 8 and 16 dpp and “Gy14” and “Poinsett 76” fruit at 16 dpp.

Supplementary Figure 2 Gompertz models and statistical analysis for cucumber experiments.
