# Supplementary figures and images for: Quantitative High-Throughput, Real-Time Bioassay for Plant Pathogen Growth in vivo
Source: Front Plant Sci. 2021 Feb 10;12:637190. doi: 10.3389/fpls.2021.637190 (PMC7902728; doi:10.3389/fpls.2021.637190)

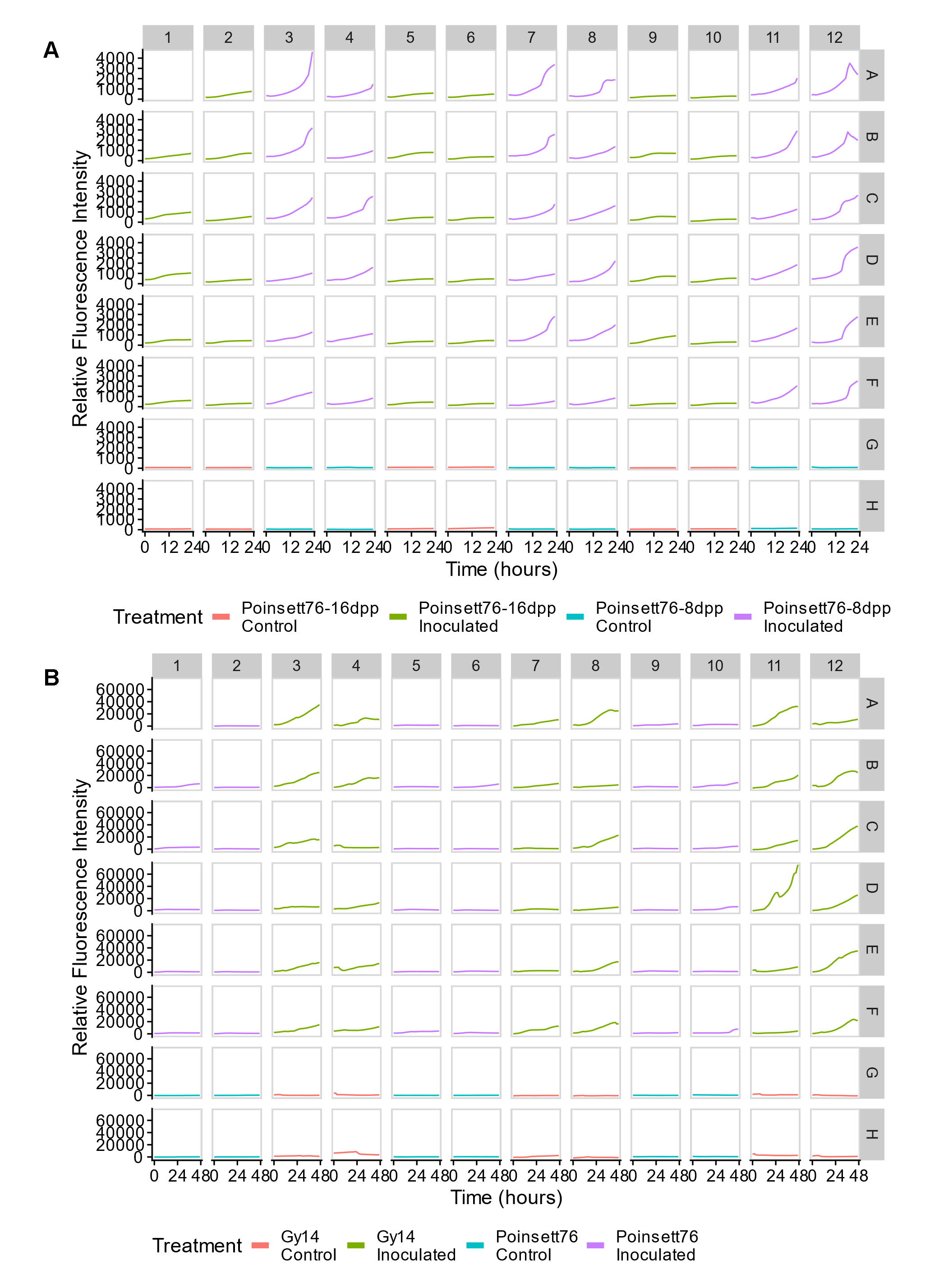

Supplement: Supplementary Figure 1 — Fluorescence intensity curves of P. capsici NY 0664-1-RFP on “Poinsett 76” fruit at 8 and 16 dpp and “Gy14” and “Poinsett 76” fruit at 16 dpp. [file Image_1.TIF]

# Gy14 vs A4-3

A

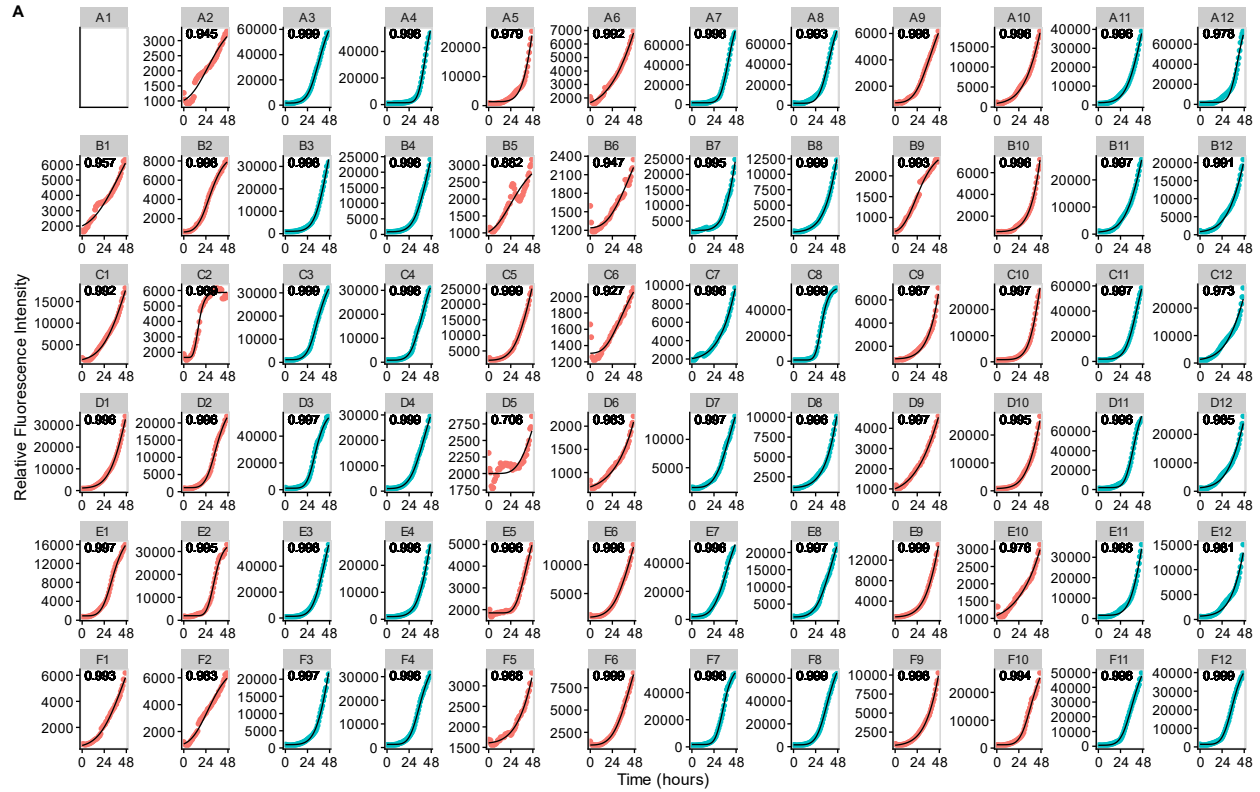

genotype • A4-3 • Gy14

B

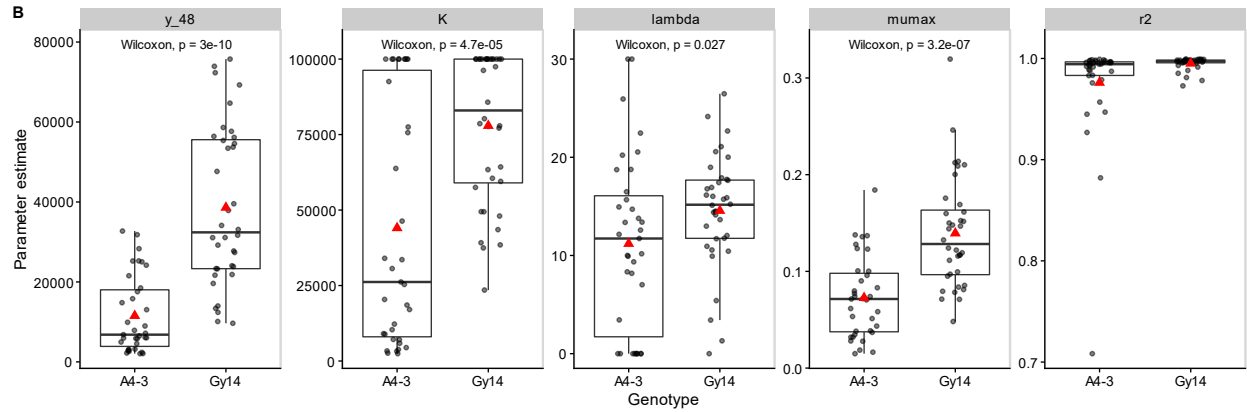

Poinsett 76 8 vs 16 dpp

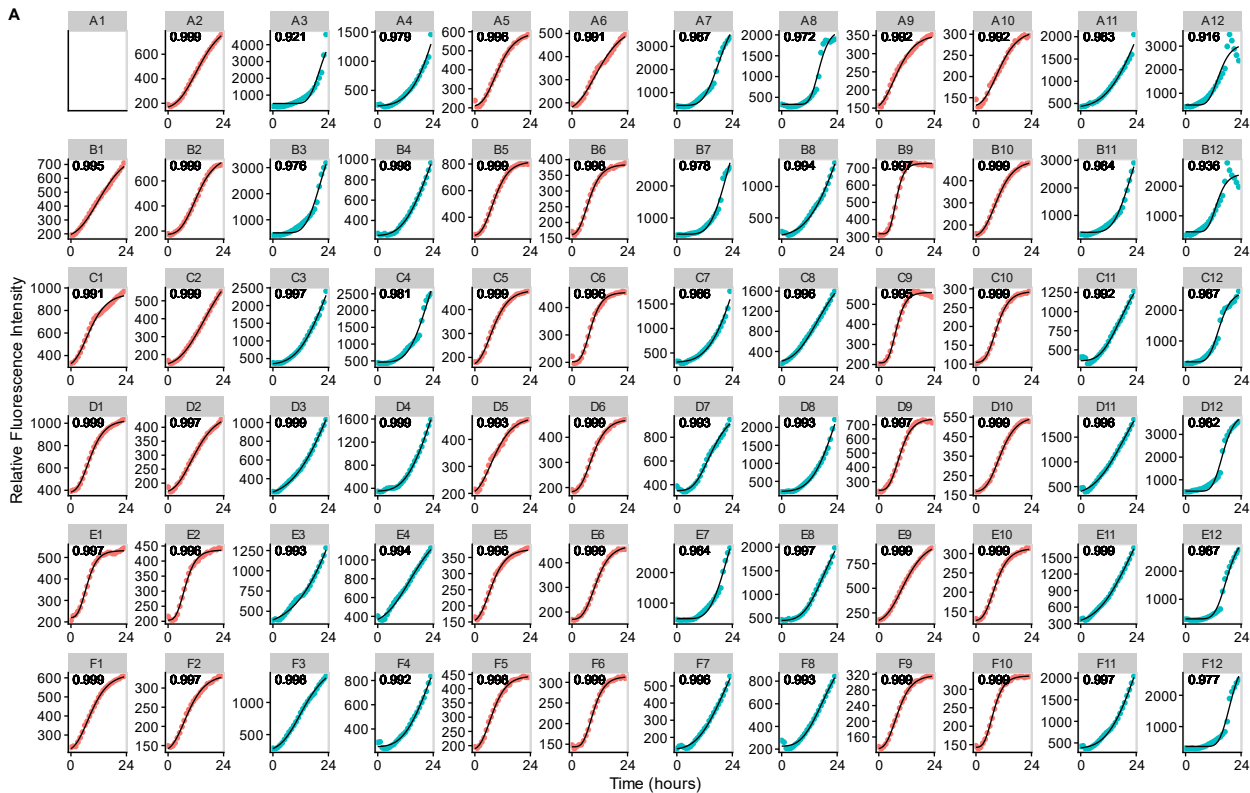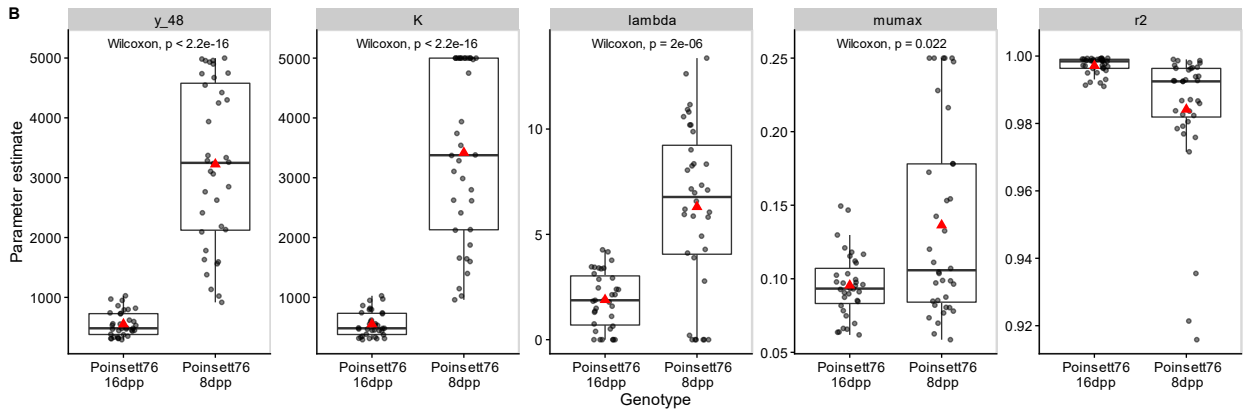

Gy vs Poinsett 76

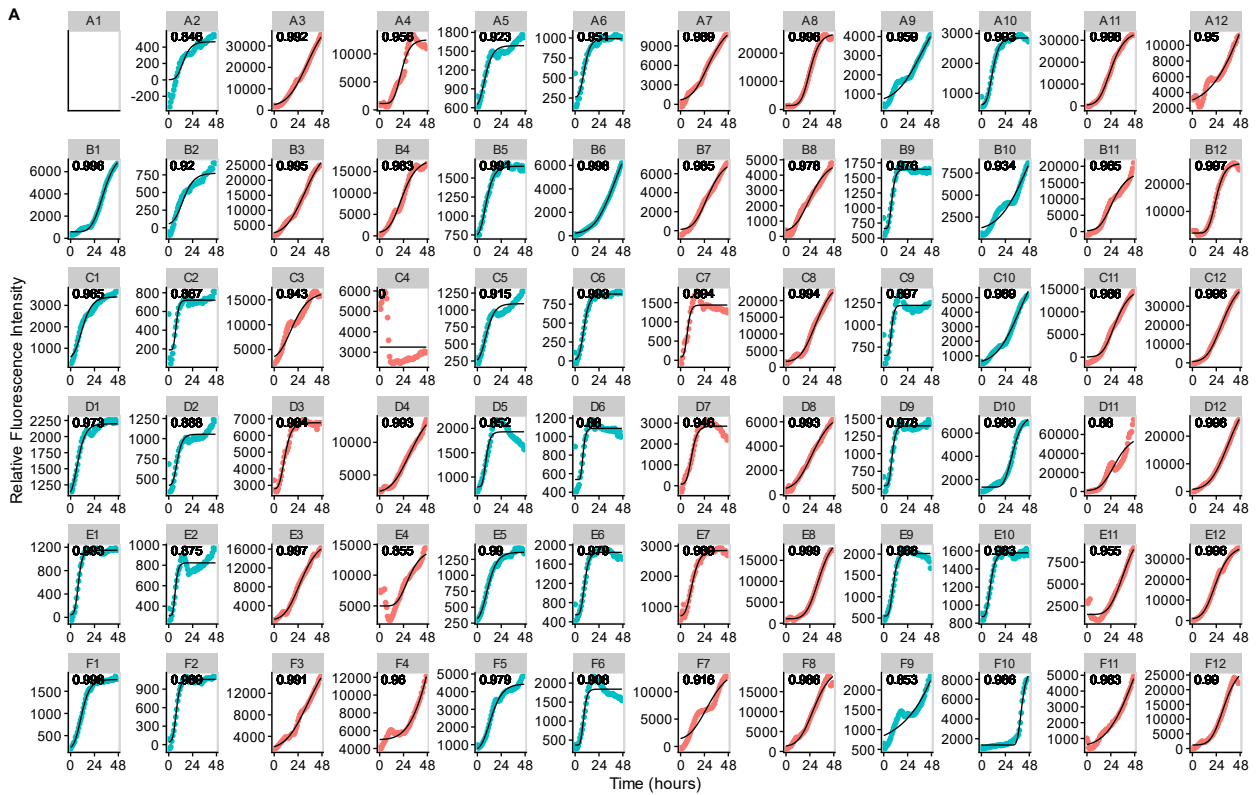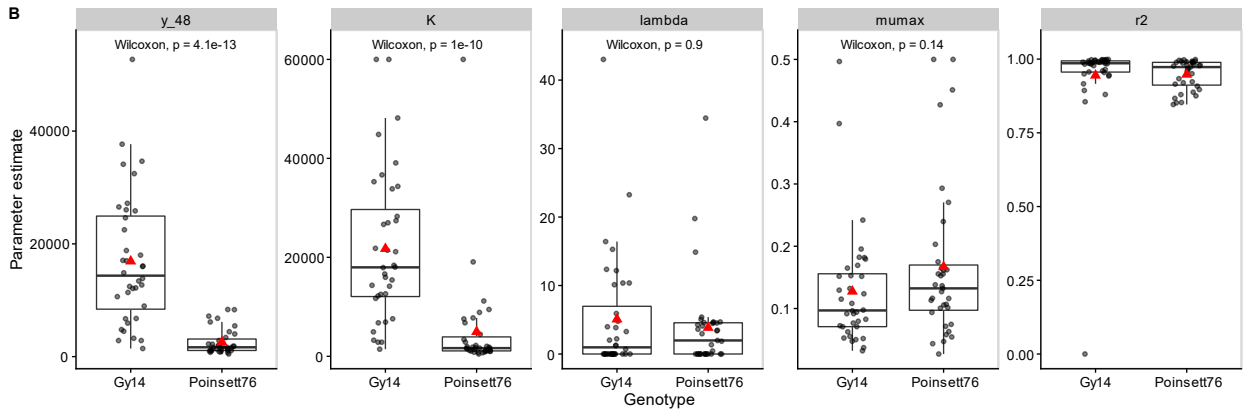

Supplement: Supplementary Figure 2 — Gompertz models and statistical analysis for cucumber experiments. [file Data_Sheet_1.PDF]
